# The Feasibility of Venovenous Extracorporeal Life Support to Treat Acute Respiratory Failure in Adult Cancer Patients

**DOI:** 10.1097/MD.0000000000000893

**Published:** 2015-05-29

**Authors:** Meng-Yu Wu, Tzu-I. Wu, Yuan-His Tseng, Wen-Chi Shen, Yu-Sheng Chang, Chung-Chi Huang, Pyng-Jing Lin

**Affiliations:** From the Department of Cardiovascular Surgery, Chang Gung Memorial Hospital and Chang Gung University, Taoyuan (M-YW, Y-HT, Y-SC, P-JL); Department of Obstetrics and Gynecology, Wan Fang Hospital, Taipei Medical University, Taipei (T-IW); Department of Hematology-Oncology (W-CS); and Department of Thoracic Medicine, Chang Gung Memorial Hospital and Chang Gung University, Taoyuan, Taiwan (C-CH).

## Abstract

Venovenous extracorporeal life support (VV-ECLS) is a lifesaving but invasive treatment for acute respiratory failure (ARF) that is not improved with conventional therapy. However, using VV-ECLS to treat ARF in adult cancer patients is controversial.

This retrospective study included 14 cancer patients (median age: 58 years [interquartile range: 51–66]; solid malignancies in 13 patients and hematological malignancy in 1 patient) who received VV-ECLS for ARF that developed within 3 months after anticancer therapies. VV-ECLS would be considered in selected patients with a P_a_O_2_/F_i_O_2_ ratio ≤70 mmHg under advanced mechanical ventilation.

Before ECLS, the medians of intubation day, P_a_O_2_/F_i_O_2_ ratio, and Sequential Organ Failure Assessment (SOFA) score were 8 (2–12), 62 mmHg (53–76), and 10 (9–14), respectively. The case numbers of bacteremia, thrombocytopenia (platelet count <50000 cells/μL), and neutropenia (actual neutrophil count <1000 cells/μL) detected before ECLS were 3 (21%), 2 (14%), and 1 (7%), respectively. After 24 hours of ECLS, a significant improvement was seen in P_a_O_2_/F_i_O_2_ ratio but not in SOFA score. Six patients experienced major hemorrhages during ECLS. The median ECLS day, ECLS weaning rate, and hospital survival were 11 (7–16), 50% (n = 7), and 29% (n = 4). The development of dialysis-dependent nephropathy predicted death on ECLS (odds ratio: 36; 95% confidence interval: 1.8–718.7; *P* = 0.01). With a median follow-up of 11 (6–43) months, half of the survivors died of cancer recurrence and the others were in partial remission.

The most prominent benefit of VV-ECLS is to improve the arterial oxygenation and rest the lungs. This may increase the chance of recovery from ARF in selected cancer patients.

## INTRODUCTION

Extracorporeal life support (ECLS) is a transformation of cardiopulmonary bypass (CPB). Equipped with miniaturized consoles, percutaneously delivered cannulae, and heparin-coated circuits, ECLS can be expeditiously administered outside the operation room and provides effective hemodynamic (venoarterial mode; VA) or ventilatory (venovenous mode; VV) support for several days.^1^ ECLS can shortly take over the pumping or ventilatory function of the injured cardiopulmonary system, and may buy some time for physicians to perform essential diagnostic and therapeutic interventions on these extremely critical patients.^2–6^ Continuous renal dialysis can also be performed on ECLS simply with a dialyzer integrated to the ECLS circuit.^7^ However, ECLS is an invasive therapy, which can increase the risk of hemorrhage.^1^ It is also a resource-demanding therapy and may impose an undue financial burden on the healthcare system if used arbitrarily.^1,8^ After weighting the therapeutic benefits against the potential risks, ECLS is principally used to treat acute cardiac or respiratory failure induced by potentially treatable or reversible etiologies including coronary artery disease, pulmonary embolism, myocarditis, and acute respiratory failure (ARF) in patients without major chronic illness.^1,9,10^ Therefore, administering ECLS to patients with malignancies may be a therapeutic controversy, as the progression of cancer can neither be intervenable or reversible. Recently, this viewpoint is challenged by reports with successful experiences in using ECLS to assist the resection of some difficult malignancies in mediastinum^11^ or to bridge patients to recovery from an advanced ARF associated with anticancer therapies.^12,13^ According to retrospective studies of the Extracorporeal Life Support Organization Registry data from 1992 to 2008, the overall survived-to-discharge rate of ECLS used for respiratory failure is 35% (n = 37 in 107) in children and 26% (n = 14/54) in adults with hematological or solid malignancy.^14^ In another retrospective study of a single institutional experience from 2000 to 2013,^15^ the survived-to-discharge rate of ECLS used for ARF in 14 adult patients with hematologic malignancy is 50%. Despite encouraging results, the heterogeneities of inclusion criteria and therapeutic strategies of ECLS among these studies still leave room for discussion. To find possible answers, an 8-year experience of ECLS in a single institution was reviewed.

## MATERIALS AND METHODS

### Study Population

From April 2006 to March 2014, a total of 583 patients received ECLS for hemodynamic support (VA mode; n = 452) or pulmonary support (VV mode; n = 131) at Chang Gung Memorial Hospital. Among the 112 adult patients (>18 years) of VV-ECLS, 14 patients had concomitant cancers and were enrolled in this retrospective study. This study was conducted in accordance with the amended Declaration of Helsinki. The ethics committee of the Chang Gung Medical Foundation approved the protocol (CGMF IRB no. 103-4427B) and waived the necessity of individual patient consent.

### Data Collection

For each patient included in this study, the following data were collected: age, characteristics of cancer and anticancer treatments, identified pathogens in body fluids (sputum or blood), organ failure scores, and results of common laboratory tests of blood (biochemistry, coagulation, cell counts, and arterial gas analysis) before and during VV-ECLS. The duration of intubation, length of VV-ECLS, and hospital days were also collected. The clinical outcomes were categorized into 3 groups (succumbed on VV-ECLS, weaned off VV-ECLS but succumbed at hospital, and weaned off VV-ECLS then survived to hospital discharge). The post-discharge status of the survivors (survived without cancer, survived with partial remission of the cancer, or died for cancer recurrence) obtained from the records of their latest visit to our outpatient clinic or emergency room. This postdischarge follow-up was ended in October 2014.

### Concerns About Patients With Active Malignancy

As shown in our previous report,^2^ the purpose of VV-ECLS is to offer prepulmonary blood gas exchange by an artificial lung to reduce the demand of oxygenation through the native lungs. With an improvement of blood oxygenation, physicians can safely re-adopt the lung protective ventilation with a small tidal volume and a decreased F_i_O_2_ to eliminate the ventilator-induced lung injuries (VILI). For ARF patients without major chronic illness, VV-ECLS was administered when adequate arterial oxygenation cannot be maintained with mechanical ventilation alone, often with P_a_O_2_/FiO_2_ ratio <70 mmHg under a FiO_2_ ≥0.8 and a positive end-expiratory pressure (PEEP) >5 cmH_2_O. For ECLS specialists, administrating this invasive therapy to cancer patients is more complicated, as this treatment may be a “futile medical care” that may cause complications rather than changing outcomes in these vulnerable patients.^16^ The oncologist-in-charge must to persuade the ECLS specialist to proceed by convincing them that the cancer itself is well controlled and the outcome may be optimistic if the patient has recovered from this episode. As all patients with advanced ARF received paralytic sedation to avoid patient-ventilator asynchrony and could not be communicated with, VV-ECLS would be exclusively delivered to patients without the “do-not-resuscitate” documents that were previously signed by themselves or by their legal representative in the index hospitalization. Patients with chronic organ failures, late-stage cancers, or abnormal brainstem reflex were also not included in the candidate pool of VV-ECLS.

### Protocol of VV-ECLS in Adult ARF

**FIGURE 1:**
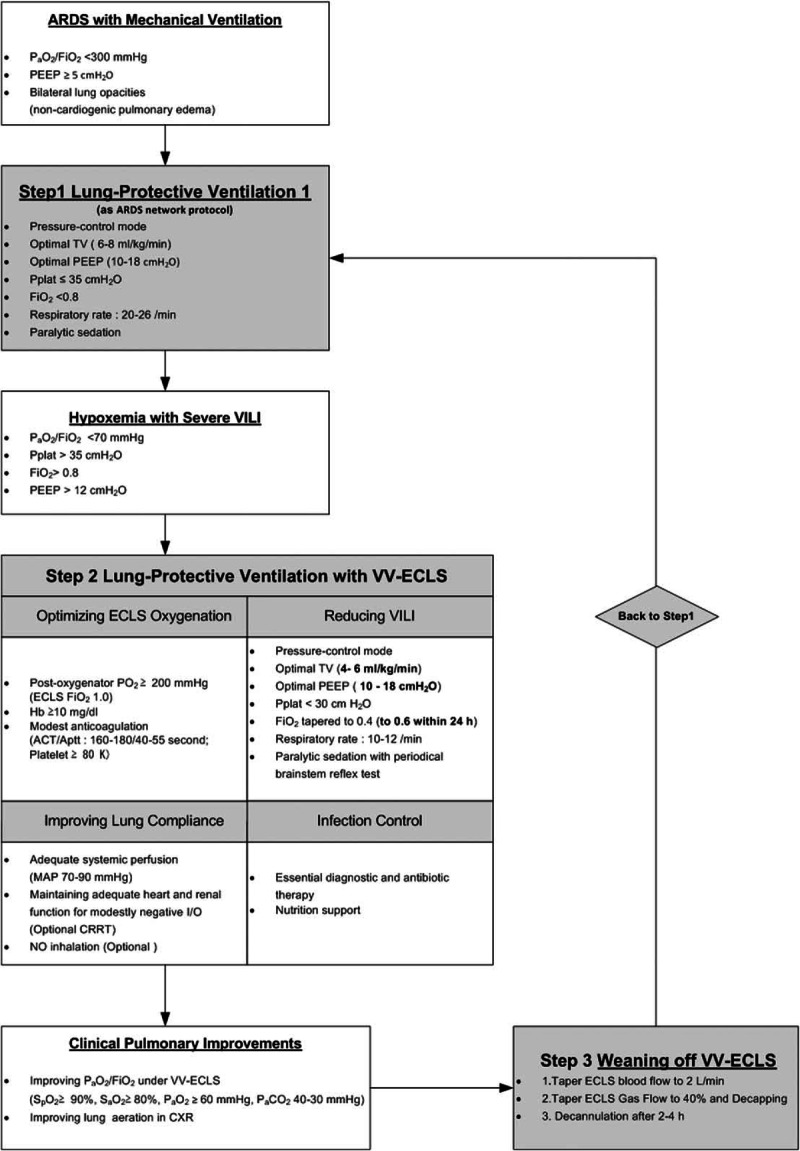
The therapeutic protocol of venovenous extracorporeal life support in adult patients with acute respiratory failure. ACT = active clotting time, aPTT = activated partial thromboplastin time, ARDS = acute respiratory distress syndrome, CRRT = continuous renal replacement therapy, CXR = chest X-ray, FiO_2_ = the fraction of inspired oxygen, Hb = hemoglobin, MAP = mean arterial pressure, NO = nitric oxide, P_a_CO_2_ = arterial carbon dioxide tension, P_a_O_2_ = arterial oxygen tension, PEEP = positive end-expiratory pressure, P_plt_ = inspiratory plateau pressure, S_p_O_2_ = pulse oximetry-detected oxyhemoglobin saturation, TV = tidal volume, VILI = ventilator-induced lung injury, VV-ECLS = venovenous extracorporeal life support.

As reported previously,^2,6,17^ we used the Capiox emergent bypass system (Terumo Inc, Tokyo, Japan) as our ECLS device. As a double lumen catheter was not available during the period of study, 2 vascular cannulae (DLP Medtronic, Minneapolis, MN; inflow: 19–23 French, outflow: 17–21 French) were used to established the femoral (in)-jugular (out) VV-ECLS via percutaneous cannulation. Figure 1 illustrates our therapeutic protocol for adult ARF in detail. Four highlighted points included: optimizing the ECLS oxygenation, reducing the VILI, improving pulmonary compliance by a modest dehydration, and controlling the infection. We routinely administered the heparin-minimized ECLS to patients presenting evidences of hypocoagulation. The common presentations of hypocoagulation include a prolonged prothrombin time (PT) with an international normalized ratio (INR) >1.2, a platelet count <80000 cells/μL, or recent episodes of spontaneous hemorrhage. In the conventional ECLS, a modest anticoagulation was maintained with an additional intravenous heparinization. The therapeutic range of the active clotting time (ACT)/activated partial thromboplastin time (aPTT) was 160–180/40–55 s. In patients with the heparin-minimized ECLS, no intravenous heparin was provided till 48 hours later. In patients with hemorrhages during ECLS, withholding heparin plus blood transfusion was the first step to achieve hemostasis. Endoscopic, angiographic, or surgical hemostasis was performed with a low threshold once transfusion alone failed to stabilize the hemorrhage. If significant improvements of pulmonary function were shown, the weaning process of ECLS was preceded under lung protective ventilation.

### Statistical Analysis

Statistical analyses were performed using SPSS for Windows (Version 15.0, SPSS, Inc, IL). Because the dataset was small, nonparametric methods including the Mann–Whitney *U* or Wilcoxon signed ranks tests was used to conduct univariate comparisons of the independent or paired continuous variables. The *χ*^2^ or Fisher exact test was used to compare the categorical variables. The multivariate logistic regression analysis was used to identify independent predictors of death on ECLS or death in hospital. The level of statistical significance was set at *P* < 0.05. The Kaplan–Meier method was used to estimate the survival from the beginning of ECLS to the patient's last medical record in our institution.

## RESULT

### Cancer Status Before ECLS

Table 1 summarizes the characteristics and the previous anticancer therapies of the 14 patients (median age: 58 years, interquartile range [IQR]: 51–66). Thirteen in the 14 patients had solid tumors, and 1 had hematological malignancy. All except 3 patients (Case 5, 9, and 12) had received surgeries for tumor resection, including liver transplantation, to cure or control the current malignancy. Two patients had recurrent cancers (Case 9 and 12) and 1 had stable distant metastasis treated with periodical chemotherapy (Case 14). The possible etiologies of ARF in the 14 patients were postoperative infections (n = 9, 8 with a complete tumor resection), tumor lysis syndrome (n = 1, Case 9) and opportunistic infections (n = 4; Case 5, 10, 12, and 14).

**TABLE 1:**
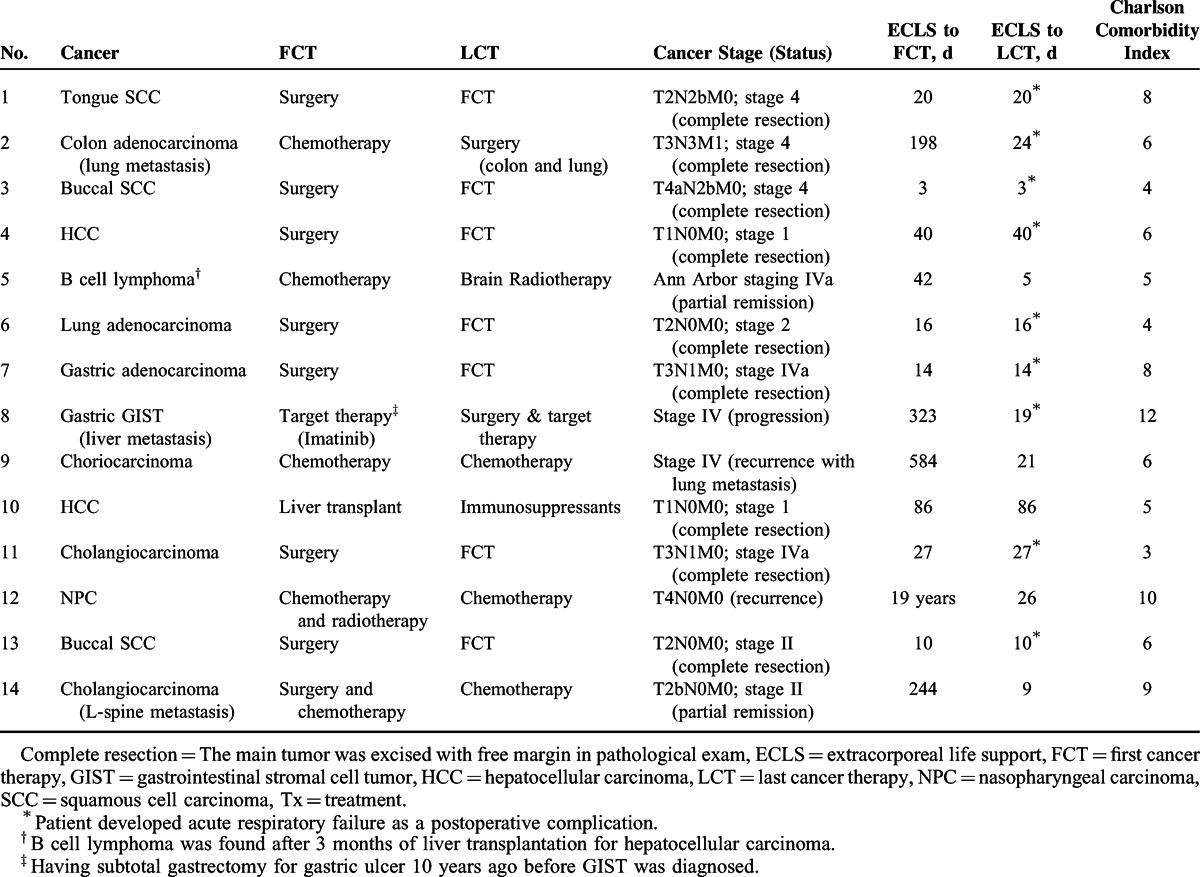
Individual Characteristics of Cancer and Associated Therapies Before ECLS

### Response to VV-ECLS and Clinical Outcomes

Table 2 summarizes the clinical and laboratory data collected before VV-ECLS. The medians of pre-ECLS intubation days, P_a_O_2_/F_i_O_2_ ratio, and Sequential Organ Failure Assessment (SOFA) score^18^ in the 14 patients were 8 (IQR 2–12), 62 mmHg (IQR 53–76), and 10 (IQR 9–14), respectively. Before VV-ECLS, 2 patients (Case 4 and 9) developed a dialysis-required acute renal failure, and the numbers of bacteremia, thrombocytopenia (platelet count <50000 cells/μL), and neutropenia (absolute neutrophil count [ANC] <1000 cells/μL) were 3 (21%), 2 (14%), and 1 (7%), respectively. Two patients (Case 5 and 9) were also treated with granulocyte colony-stimulating factor (GCSF) before VV-ECLS. Tables 3–5 summarize the clinical, laboratory, and ventilatory data collected during VV-ECLS. The P_a_O_2_/F_i_O_2_ ratio was significantly improved (from 62 to 101 mmHg, *P* = 0.02) immediately after the administration of VV-ECLS, and the improvement continued for the next 24 hours (from 101 to 167 mmHg, *P* = 0.002). The pulmonary protective strategy (Pplt ≤30 cmH2O with FiO_2_ ≤0.6) was achieved in 4 patients within 24 hours after VV-ECLS. The median platelet count soon decreased after VV-ECLS, from 115000 to 99000 cells/μL (*P* = 0.01). A parallel increase of white cell count and ANC was seen in 9 patients soon after VV-ECLS, but the incidence of neutropenia increased to 13% (n = 2, Case 5 and 9). The medians of the vasoactive inotropic score (VIS)^19^ and SOFA score were not significantly changed after 24 hours of VV-ECLS (before versus after in VIS: 10 vs 8 μg/kg/min, *P* = 0.88; in SOFA score: 10 vs 11, *P* = 0.78). Six patients experienced major hemorrhages, and 7 patients had a dialysis-dependent nephropathy during ECLS. The median ECLS duration, ECLS weaning rate, and hospital survival were 11 (IQR: 7–16) days, 50% (n = 7), and 29% (n = 4), respectively. Three of the 4 survivors were patients developing postoperative ARF after a complete tumor resection (Case 1, 3, and 7), and the other one was the patient having an opportunistic viral pneumonia after chemotherapy (Case 14). None of the patients survived if they had a progressive or recurrent cancer, a GCSF-required neutropenia, or a dialysis-dependent nephropathy during this episode. The multivariate logistic regression revealed that the development of dialysis-dependent nephropathy predicted death on ECLS (odds ratio: 36; 95% confidence interval: 1.8–718.7; *P* = 0.01; c-index = 0.86). With a median follow-up of 11 (IQR: 6–43) months after surviving to hospital discharge, 2 (Case 1 and 7) of the 4 survivors died of recurrent cancers and the others survived in partial remission.

**TABLE 2:**
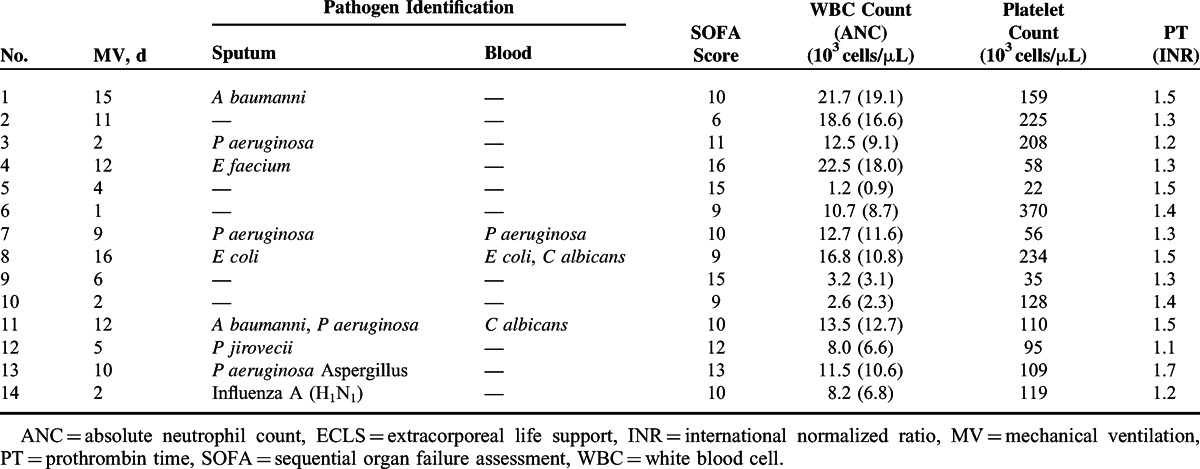
Clinical and Laboratory Information Before Venovenous ECLS

**TABLE 3:**
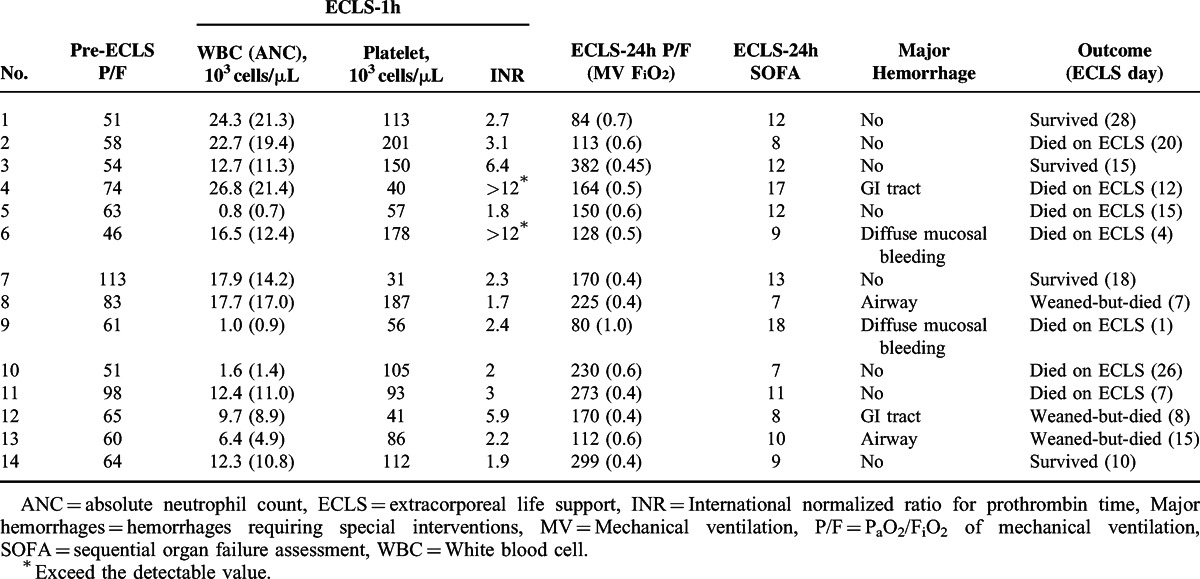
Clinical and Laboratory Information During Venovenous ECLS

**TABLE 4:**
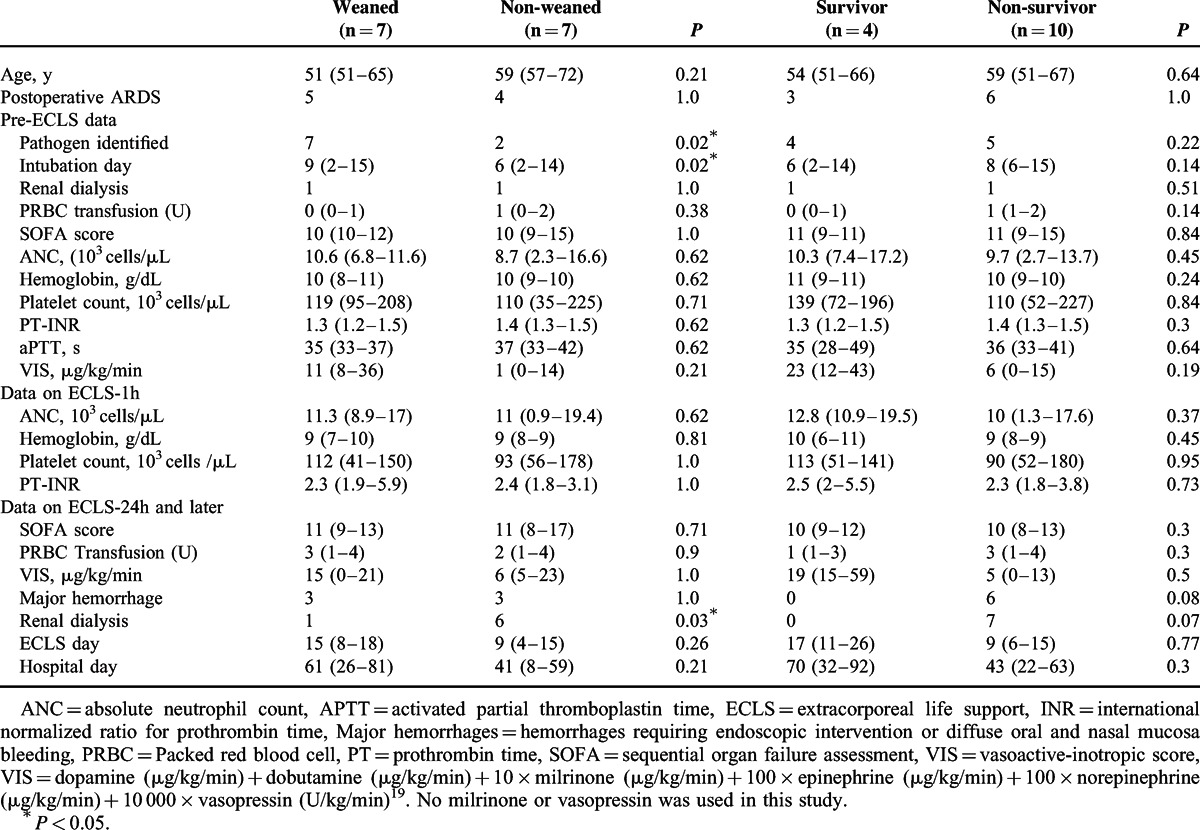
Comparisons of Demographic and Laboratory Variables Between Groups With Different Outcomes of Venovenous ECLS

**TABLE 5:**
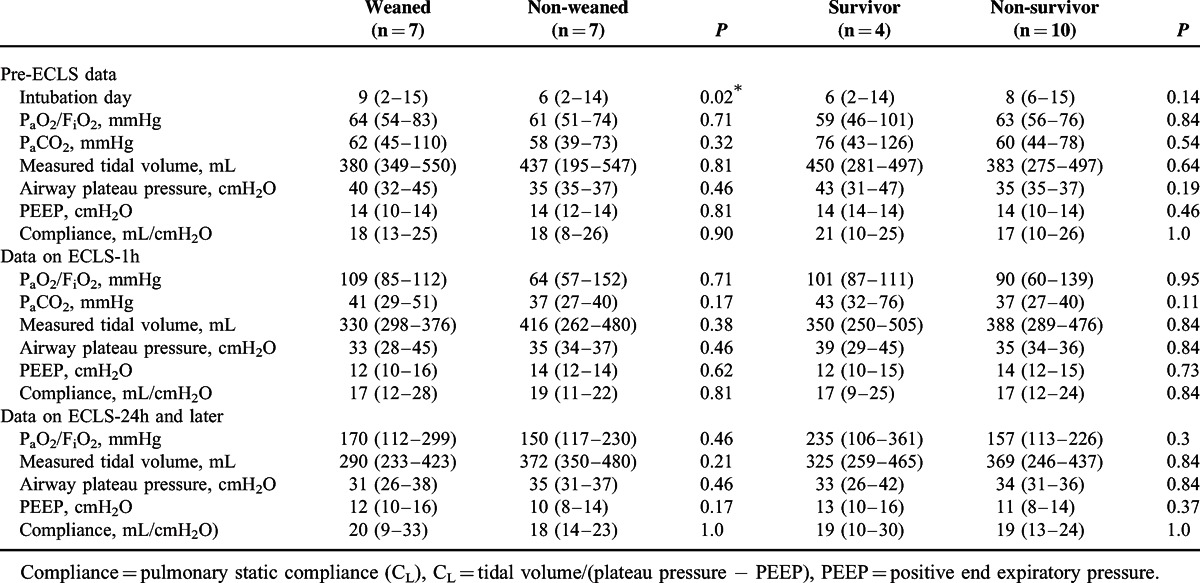
Comparisons of Common Ventilatory Parameters Between Groups With Different Outcomes of Venovenous ECLS

## DISCUSSION

This study aimed to investigate the feasibility of using VV-ECLS to treat ARF in adult cancer patients. As ECLS is a life support in essence, it is naturally for physicians using ECLS to bail out their patients of cardiopulmonary crises induced by miscellaneous etiologies, including cancers and anticancer therapies. The incidence of ARF in cancer patients is around 10% to 50%.^20^ Invasive ventilation with endotracheal intubation is often the last resort therapy for severe ARF in these patients and yields a high mortality rate of around 60% to 70%.^20–22^ As cancer patients are not traditionally the key population of interest in critical care medicine, it is not surprising that this mortality rate remains unchanged across the last decade, although some therapeutic breakthroughs of ARF have been made with ECLS during this time.^10,23^ In fact, with an increasing ability to inhibit tumor growth, modern anticancer therapies do change the progression of cancers to a relapsing-and-remitting cycle and prolong life span of some cancer patients.^16^ This prolongation of life span may also increase the risk of ARF in cancer patients. With an increasing experience of ECLS, it may be time to cautiously assess the indications and therapeutic benefits of ECLS for ARF in cancer patients. To simplify the interpretations of outcomes, this study enrolled patients treated with VV-ECLS exclusively, as VV-ECLS is the major strategy of ECLS used to treat adult ARF.^24^

The criterion of patient selection is often the source of controversies in such reports.^16^ Patients of this study were divided into the postoperative ARF group and the non-postoperative ARF group. VV-ECLS was delivered directly to the postoperative ARF group because they may generally have a similar risk of hemorrhage during ECLS as the non-cancer patients after major operations. Doubts about futile medical care were considered and demanded more discussions in the non-postoperative ARF group. Using a standardized protocol of VV-ECLS that yields a hospital survival rate of 70% for post-traumatic ARF,^2^ this study obtained a hospital survival rate of 29% for ARF in adult cancer patients. As expected, the postoperative ARF group achieved a better hospital survival rate (33%, 3/9) than the non-operative ARF group (20%, 1/5). The cause of this discrepancy of survival rates was not exactly known, although some of the patients in the non-operative group were neutropenic and had an unknown source of infection.

**FIGURE 2:**
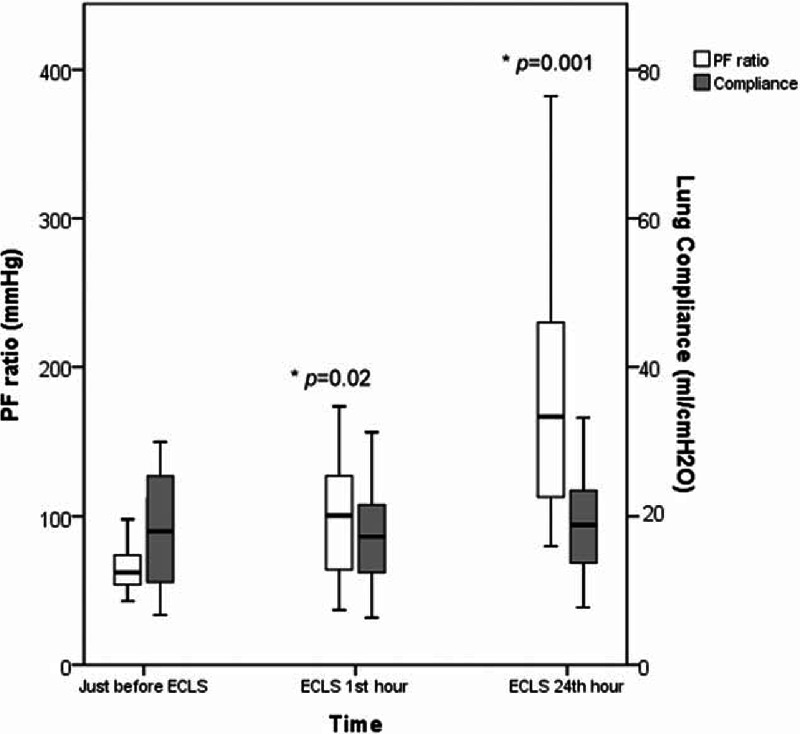
The box plots of the change of P_a_O_2_/F_i_O_2_ ratio and lung compliance (static) with time.

Tables 4 and 5 demonstrate the laboratory and ventilatory influences of VV-ECLS on these patients. Without an improved compliance of the native lungs, all patients still experienced an expeditious improvement in blood gas exchange soon after the administration of VV-ECLS, as showed in Figure 2. A decreased platelet count with an extremely prolonged PT/aPTT was also found in these patients, as discovered in our previous report.^2,25^ However, it is interesting that leukocytosis was only seen in some but not in all patients in this cohort. Leukocytosis reflects an immediate immune response to the violent blood-surface interaction initiated in all forms of extracorporeal circulation (CPB, ECLS, and hemodialysis).^26^ In our previous report of using ECLS to treat adult myocarditis,^17^ all patients showed an increased leukocytosis immediately and the median increase of leukocyte in the first 24 hours of ECLS is 79% (from 8200 to 14700 cells/μL). The scale of this increment should be >79% in fact because the volume of distribution is increased in patients connected to ECLS. Therefore, owing to an increased volume of distribution and continuous blood consumption, we may reasonably assume that ECLS can further increase the severity of neutropenia, thrombocytopenia, and even anemia in patients with a severely compromised immune-hematopoietic system. This may increase the risk of hemorrhage on ECLS and also the demand of blood transfusion, which may further amplify the injuries to the pulmonary and the immune-hematopoietic systems.^13,15,27^ Nonetheless, a severe neutropenia or pancytopenia is not necessary a predictor of death on ECLS,^27^ and it may be corrected with allogenic hematopoietic stem cell transplantation on ECLS.^28^ The type of the pre-existing infection and the number of organ dysfunction also have substantial impacts on the outcomes of ECLS.^25,28–30^ An insufficient infection control due to a lack of identifiable pathogens is also a therapeutic difficulty in this patient population.^21^ The present study found that the patients with a defined pathogen of infection showed a higher possibility to wean VV-ECLS than the patients without (weaning rate: 78% vs 0, *P* = 0.02). The development of dialysis-dependent nephropathy on ECLS reflects a progression of sepsis,^31^ and mortality is inevitably if the sepsis cannot be treated effectively, even the patient can be barely weaned from ECLS.^25,32^

While treating patients with ECLS, the top-priority task of the ECLS specialist is to choose a proper mode of ECLS to meet the patient's need and to maintain a sound hemodynamics on the support. Theoretically, VV-ECLS should have little impact on hemodynamics. However, in our experiences, the patient's demand of vasoactive-inotropic agents was often fluctuated during the first 24 hours of VV-ECLS. They might develop hypotension during or soon after cannulation of VV-ECLS as a result of blood loss or supraventricular arrhythmias induced by right atrial catheterization. The demand of vasopressors was often temporarily increased to correct this hypotension but could be reversed after adequate volume replacement and readopting the lung-protective ventilation, as this ventilation could reduce the intrathoracic pressure and improve the output of right ventricle (RV).^33^ However, ventricular arrhythmia even asystole might occur immediately after the start of VV-ECLS, especially in patients demanding high levels of vasoactive-inotropic agents (often combined with a high translung pressure) to maintain an acceptable hemodynamics before VV-ECLS. It may be explained as an acute RV failure resulting from an abrupt loss of its preload from the right atrium that is also rapidly drained to the ECLS circuit.^34^ Thus, to reduce the chance of the immediate shock, VA-ECLS rather than VV-ECLS should be considered in patients with an advanced demand of vasopressors.^33^

The limitations of this study are its retrospective design and the small number of cases involved. A full assessment of the therapeutic impacts of ECLS on cancer patients with ARF was not achieved because only the patients treated with VV-ECLS were included. Further prospective and collaborative studies involving a large population and an integrated protocol of continuing anticancer therapies during ECLS are necessary to optimize the analysis of the therapeutic effects of ECLS on cancer patients with ARF.

## CONCLUSION

The most prominent therapeutic benefit of VV-ECLS is to improve the arterial oxygenation and rest the lungs. This might buy selected cancer patients time to fight infections, with the cost of an increased risk of hemorrhage and demand of blood transfusion. However, VV-ECLS was not so feasible to treat ARF in cancer patients with a recurrent or progressive malignancy, a severe neutropenia, or a dialysis-required acute renal failure.
